# High NRF2 level mediates cancer stem cell-like properties of aldehyde dehydrogenase (ALDH)-high ovarian cancer cells: inhibitory role of all-*trans* retinoic acid in ALDH/NRF2 signaling

**DOI:** 10.1038/s41419-018-0903-4

**Published:** 2018-08-30

**Authors:** Donghyeok Kim, Bo-hyun Choi, In-geun Ryoo, Mi-Kyoung Kwak

**Affiliations:** 1 Department of Pharmacy, Graduate School of the Catholic University of Korea, 43 Jibong-ro, Bucheon, Gyeonggi-do 14662 Republic of Korea; 2 Integrated Research Institute for Pharmaceutical Sciences, The Catholic University of Korea, 43 Jibong-ro, Bucheon, Gyeonggi-do 14662 Republic of Korea; 3 College of Pharmacy, The Catholic University of Korea, 43 Jibong-ro, Bucheon, Gyeonggi-do 14662 Republic of Korea

## Abstract

Aldehyde dehydrogenase 1A1 (ALDH1A1) is one of cancer stem cell (CSC) markers, and high ALDH1 expression has been related to drug resistance and facilitated tumor growth. In this study, we investigated the potential involvement of nuclear factor erythroid 2-like 2 (NFE2L2/NRF2) in CSC-like properties of ALDH-high ovarian CSCs. Our experimental system, ALDH1A1-high (ALDH-H) subpopulation, was isolated and stabilized using doxorubicin-resistant ovarian cancer A2780 cells. ALDH-H exerted CSC-like properties such as drug resistance, colony/sphere formation, and enhanced tumor growth along with high levels of CSCs markers compared to ALDH1A1-low (ALDH-L). Levels of NRF2 and subsequent target genes substantially increased in ALDH-H cells, and the increase in ALDH1A1 and p62 was associated with NRF2 upregulation. *ALDH1A1*-silencing blocked increases in NRF2, drug efflux transporters, and p62, along with CSC markers in ALDH-H cells. The inhibition of *p62*, which was elevated in ALDH-H, suppressed NRF2 activation. High NRF2 level was confirmed in the ALDH1-high subpopulation from colon cancer HCT116 cells. The functional implication of NRF2 activation in ovarian CSCs was verified by two experimental approaches. First, CSC-like properties such as high CSC markers, chemoresistance, colony/sphere formation, and tumor growth were significantly inhibited by *NRF2*-silencing in ALDH-H cells. Second, all-*trans* retinoic acid (ATRA) suppressed ALDH1 expression, inhibiting NRF2 activation, which led to the attenuation of CSC-like properties in ALDH-H cells but not in ALDH-L cells. These results provide insight into the molecular basis of the ALDH1A1-mediated development of CSC-like properties such as stress/treatment resistance, and further suggest the therapeutic potential of ATRA in ALDH-high ovarian CSCs.

## Introduction

A small subpopulation of tumor cells, called cancer stem cells (CSCs) or tumor-initiating cells (TICs), are implicated in tumor initiation and propagation^1^. CSCs were initially demonstrated in hematopoietic cancer and shown that they could be isolated from several human malignancies such as brain, breast, and colon tumors^2–5^. CSCs exhibit several characteristic properties, including enhanced self-renewal capacities, recurrence, and chemoresistance of tumor cells^6,7^. Elevated expressions of antioxidant enzymes such as superoxide dismutase-2 (SOD2) and glutathione peroxidase-1 (GPX1); drug efflux transporters such as breast cancer resistance protein (BCRP); and DNA repair enzymes contribute to therapy resistance and facilitated survival of CSCs^8^. Based on experimental and clinical evidence, several cell surface markers such CD44, CD133, and CD24 are used for the detection and isolation of CSCs from tumor tissues and cancer cell lines^9,10^.

High enzymatic activity of aldehyde dehydrogenase (ALDH) is one of CSC hallmarks^11,12^. ALDHs are involved in the oxidation of aldehydes to the corresponding carboxylic acids, including retinoic acid. The linkage between high ALDH expression and CSC-like properties of various cancers is supported by multiple lines of in vitro and clinical evidence. A subpopulation of ALDH-high prostate cancer cells isolated using the Aldefluor assay showed increased clonogenic potential and migration capacity compared to ALDH-low cancer cells^13^. ALDH1 overexpression was strongly associated with poor clinical outcomes of prostate and breast cancer patients^14,15^. A meta-analysis of 1258 ovarian cancer patients revealed high ALDH expression was correlated with decreased overall survival^16^. Of note, high ALDH expression showed a strong association with therapy resistance. Ovarian cancer patients with high ALDH1A1 expression displayed a diminished response to platinum-based chemotherapy^17^. ALDH1-positive CSC-like cells were enriched in ovarian tumors following the taxane/platinum-based therapy^18^. In line with these, ALDH1 inhibition reduced chemoresistance in head and neck cancer, and effectively blocked the proliferation and survival in ovarian cancer spheroids^19,20^. In drug-resistant ovarian cancer cell lines, high expression of BCRP and multidrug resistance protein 1 (MDR1) was accompanied by ALDH1A1 overexpression, and the inhibition of ALDH activity reduced drug efflux transporter expression, leading to sensitization to chemotherapy^21^. However, there is insufficient evidence for the molecular role of ALDH1 in CSC-like properties, including the increased drug efflux transporters and enhanced tumorigenicity.

The anticancer effect of retinoic acid is attributed to the regulation of gene expression that results in the modulation of cell differentiation, proliferation, and apoptosis^22^. All-*trans* retinoic acid (ATRA) is used for the treatment of acute promyelocytic leukemia with high remission rates^23^. Additionally, retinoic acid has been found to inhibit CSC properties and chemoresistance in several types of solid tumors. Retinoic acid treatment induced differentiation of glioblastoma stem cells, which led to the loss of CSC marker expression and the retardation of tumor growth through Notch signaling inhibition^24^. ATRA treatment repressed ALDH expression and increased the cytotoxic effect of 4-hydroperoxycyclophosphamide in lung cancer cells^25^. Ovarian CSCs were sensitized to platinum chemotherapy when retinoic acid was combined with cisplatin^26^.

Nuclear factor erythroid 2-like 2 (NFE2L2), also known as NRF2, is a key transcription factor for the cytoprotective response to oxidative and electrophilic stress. Under the oxidative stress condition, NRF2 dissociates from its molecular inhibitor Kelch-like ECH-associating protein 1 (KEAP1), and translocates into the nucleus. Then, NRF2 binds to the antioxidant response element (ARE) in the regulatory region of its target genes to induce their expression^27^. NRF2 target genes include NAD(P)H quinone oxidoreductase-1 (*NQO-1*), aldo-keto reductase 1C1 (*AKR1C1*), GSH generating enzymes, and drug efflux transporters such as BCRP^28^. The regulation of NRF2 activity through additional noncanonical pathways has been identified: p62, which was discovered as an autophagy linker protein, induces NRF2 activation by competitive binding to the KEAP1 protein and autophagic degradation of KEAP1^29,30^. Although NRF2 exhibits a wide array of beneficial effects in normal cells, its high activity has been associated with unfavorable tumor phenotypes. NRF2 expression is often enhanced in several tumor types, including lung, breast, colon, and ovarian cancer^31^, and high NRF2 level plays a critical role in tumor cell growth and chemoresistance by elevating reactive oxygen species (ROS) inhibiting enzymes and drug efflux transporters^27^. Recent studies have indicated that NRF2 signaling is engaged in CSC-like properties of several types of cancer cells. *NRF2*-knockdown inhibited the self-renewal capacity of glioma stem cells^32^. NRF2 signaling is activated in spheroid cultured breast and colon cancer cells, and high NRF2 activity in these CSC-enriched systems was responsible for anticancer resistance and facilitated spheroid growth^33,34^. In this study, we investigated the potential role of NRF2 signaling in CSC-like properties of ALDH1-enriched ovarian CSCs. We also evaluated the effects of ATRA on NRF2 activity and CSC-like properties in these cells.

## Results

### Doxorubicin-resistant ovarian cancer A2780DR cells possess high population of ALDH1-positive cells

**Fig. 1:**
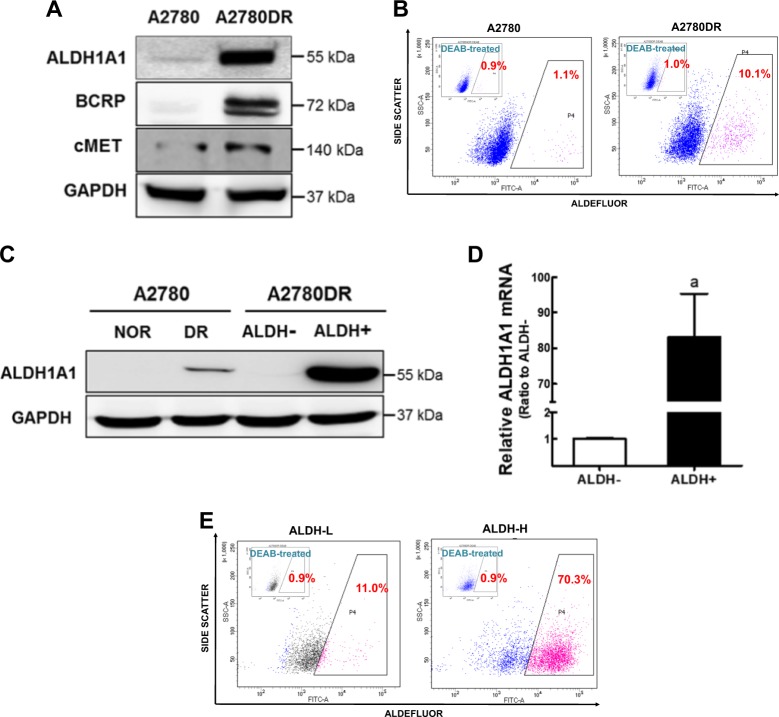
A2780DR cells show elevated ALDH1A1 levels. **a** Protein levels of ALDH1A1 and BCRP were determined in the parental A2780 cell line and doxorubicin-resistant A2780 cell line (A2780DR) by using western blot analysis. **b** ALDH1 activity was determined using the Aldefluor assay system in A2780 and A2780DR cells. ALDH1 inhibitor DEAB-treated cells were used as a negative control. Cell population with Aldefluor-derived fluorescence intensity was analyzed using flow cytometry. Similar results were obtained in 3–4 independent experiments. **c** Protein levels of ALDH1A1 were determined in ALDH-negative (ALDH−) and ALDH-positive (ALDH+) cell populations isolated from A2780DR. **d** Transcript levels for *ALDH1A1* in ALDH− and ALDH+ cells using RT-PCR analysis. Values represent the mean ± standard deviation (SD) of three experiments. ^a^*P* < 0.05 compared to the ALDH-L group. **e** ALDH enzymatic activity was assessed using the Aldefluor assay system in the established ALDH1-low A2780DR cell line (ALDH-L) and ALDH1-high A2780DR cell line (ALDH-H). Cell populations with Aldefluor-derived fluorescence intensity were analyzed using flow cytometry. Similar blots were obtained in three independent experiments (**a** and **c**)

A doxorubicin-resistant A2780DR cell line was established by maintaining ovarian cancer A2780 cells under the presence of doxorubicin^35^. A2780DR cells exhibited higher resistance to doxorubicin treatment than the parental A2780 cells (Supplementary Fig. S1), and our microarray analysis performed in the previous study^36^ revealed that *ALDH1A1* was the second highest gene to increase in A2780DR compared to A2780 (Table 1). Western blot analysis showed that the protein level for ALDH1A1 was higher in A2780DR than that in parental A2780 (Fig. 1a). Levels of BCRP and c-MET were also high in A2780DR, which confirms our previous observation^36^. Flow cytometry showed high ALDH1A1 levels in A2780DR due to an increase of ALDH1-positive (ALDH+) cell populations. In Aldefluor staining (Fig. 1b), A2780DR cells showed higher ALDH+ cell populations (10.1%) than A2780 (1.1%). When this ALDH+ cell population was isolated from A2780DR cells using a cell sorter, ALDH1A1 level in the ALDH+ population was substantially higher than those in the A2780DR and ALDH-negative (ALDH−) cell subpopulations from A2780DR (Fig. 1c).Transcript levels for *ALDH1A1* were 83-fold higher in ALDH+ compared to that in ALDH− (Fig. 1d). Isolated ALDH+ cells were maintained in normal medium for more than 2 months, and this stable cell line (ALDH1-high; ALDH-H) was shown to possess more than 70% ALDH+ cells (Fig. 1e). The stably established ALDH− cell line (ALDH1-low; ALDH-L) from A2780DR contained 11% of ALDH+ cells (Fig. 1e).

**Table 1:** Elevated gene expression in doxorubicin-resistant A2780DR when compared to A2780 in microarray analysis. Gene name is indicated in the order of fold change of mRNA level of A2780DR to that of A2780

Rank	Gene name	Description	Fold change
1	*ABCG2/BCRP*	ATP-binding cassette, sub-family G (WHITE), member 2	44.714
2	*ALDH1A1*	Aldehyde dehydrogenase 1 family, member A1	29.714
3	*ELAVL2*	ELAV (embryonic lethal, abnormal vision, Drosophila)-like 2 (Hu antigen B)	23.836
4	*CRABP1*	Cellular retinoic acid binding protein 1	18.102
5	*SPP1*	Secreted phosphoprotein 1 (osteopontin, bone sialoprotein I, early T-lymphocyte activation 1)	16.595
6	*SEMA6D*	Sema domain, transmembrane domain, and cytoplasmic domain	13.897
7	*SLC30A8*	Solute carrier family 30 (zinc transporter), member 8	13.777
8	*DCN*	Decorin	10.450
9	*LUM*	Lumican	10.310
10	*NLGN1*	Neuroligin 1	10.240

### ALDH-H cells demonstrate CSC-like properties

**Fig. 2:**
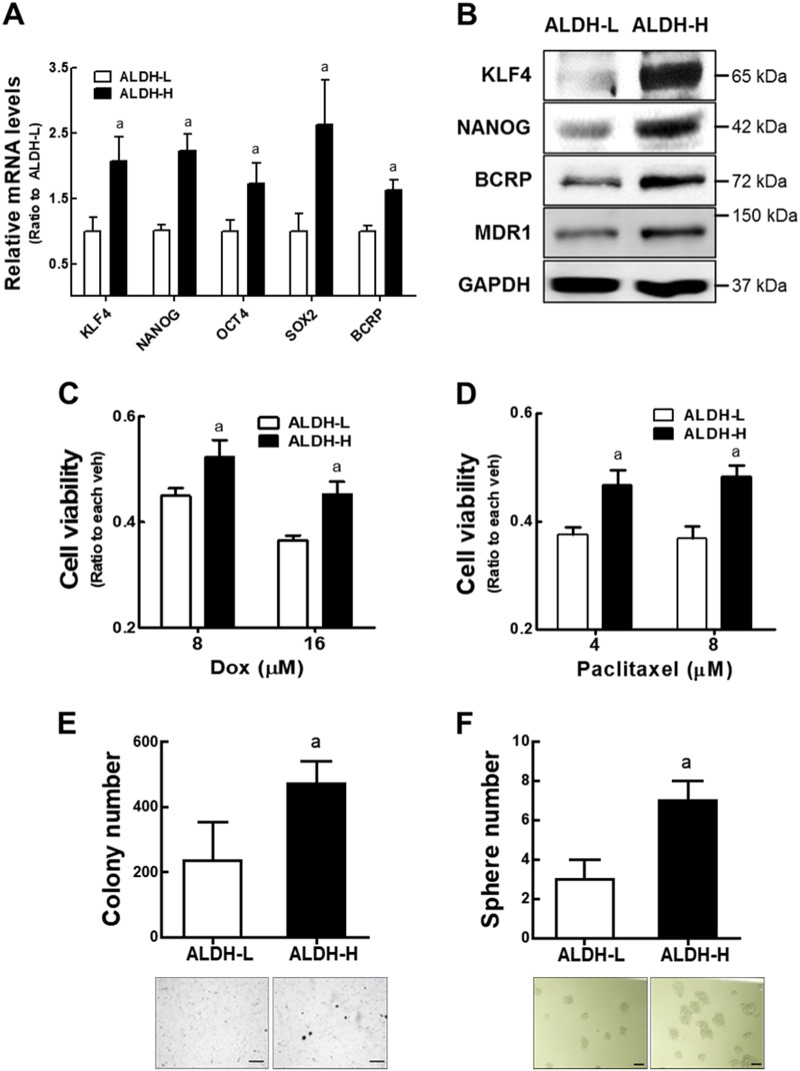
ALDH-H cells display CSC-like properties. **a** Transcript levels for *KLF4, NANOG, OCT4, SOX2*, and *BCRP* were assessed in ALDH-L and ALDH-H cells using RT-PCR analysis. Values represent the mean ± SD of three experiments. **b** Western analysis of KLF4, NANOG, BCRP, and MDR1 was carried out in ALDH-L and ALDH-H cells. Similar blots were obtained in three independent experiments. **c**, **d** Cell viability was monitored after incubation with doxorubicin (**c**) or paclitaxel (**d**) for 24 h in ALDH-L and ALDH-H cells. Values represent the mean ± SD from 8 to 10 sampled wells. ^a^*P* < 0.05 compared to the vehicle-treated group. Similar results were obtained in 2–3 independent experiments. **e** Soft agar colony formation was assessed in ALDH-L and ALDH-H cells. Values represent the mean ± SD from three dishes. **f** Sphere formation capacity was assessed after 3 days of sphere culture of ALDH-L and ALDH-H cells. Number of spheres over 100-μm diameter was counted using image processing ToupView software (×100 magnification). Scale bar = 100 μm. Values represent the mean ± SD from three independent experiments. ^a^*P* < 0.05 compared to the ALDH-L group (**e**, **f**)

In order to explore the CSC properties in ALDH+ cancer cell population, we monitored expression of CSC markers in ALDH-H. Transcripts and corresponding protein levels of CSC markers such as kruppel-like factor 4 (KLF4) and NANOG, and drug efflux transporters such as BCRP and MDR1 were relatively high in ALDH-H cells when compared to ALDH-L cells (Fig. 2a, b). ALDH-H cells were more resistant to doxorubicin or paclitaxel treatment than ALDH-L cells (Fig. 2c, d). The colony formation capacity was significantly increased in ALDH-H cells (Fig. 2e). Similarly, ALDH-H cells showed a significant increase in sphere-forming capacity compared to ALDH-L cells (Fig. 2f). These results show that high ALDH1 expression is correlated with the expression of CSC markers, drug efflux transporters, and CSC-like properties in ovarian cancer cells.

### NRF2 signaling is activated in ALDH-H cells

**Fig. 3:**
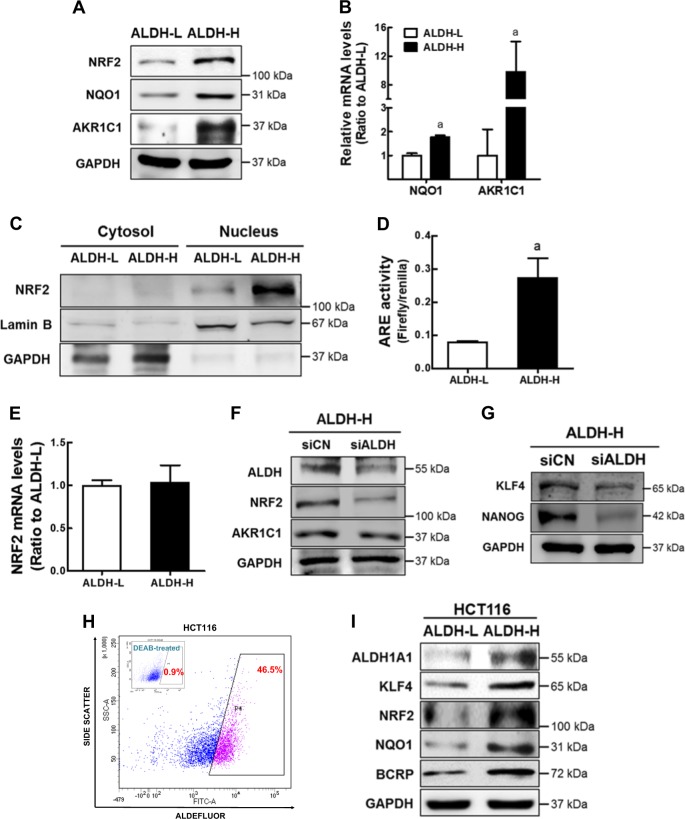
NRF2 signaling is activated in ALDH-H cells. **a** Protein levels of total cellular NRF2, NQO1, and AKR1C1 were determined in ALDH-L and ALDH-H cells using western blot analysis. **b** *NQO1* and *AKR1C1* transcript levels were monitored in ALDH-L and ALDH-H cells. ^a^*P* < 0.05 compared to the ALDH-L group. **c** Cytosolic and nuclear levels of NRF2 were assessed in ALDH-L and ALDH-H cells. Lamin B and GAPDH were determined as loading controls of nuclear and cytoplasmic proteins, respectively. **d** NRF2 transcriptional activity was monitored in ALDH-L and ALDH-H cells by measuring ARE-driven luciferase activity. ^a^*P* < 0.05 compared to the ALDH-L group. **e** *NRF2* transcript levels were assessed in ALDH-L and ALDH-H cells. **f** ALDH1A1-specific siRNA (siALDH) or nonspecific control RNA (siCN) was transfected into ALDH-H cells, and protein levels of ALDH1A1, NRF2, and AKR1C1 were determined. **g** Protein levels of KLF4 and NANOG were determined in siCN and *ALDH1A1*-silenced ALDH-H cells (siALDH). **h** ALDH activity was determined in colon cancer HCT116 cells using Aldefluor assay system. Cell population (45.5%) with strong Aldefluor-derived fluorescence intensity was isolated from HCT116 using a flow cytometry cell sorter. **i** Protein levels of ALDH1A1, KLF4, BCRP, NRF2, and NQO1 were determined in ALDH-low (ALDH-L) and ALDH-high (ALDH-H) HCT116 cell populations. Similar blots were obtained in three independent experiments (**a**, **c**, **f**, **g**, and **i**). Values are means ± SD from three experiments (**b**, **d**, and **e**)

We previously observed that high NRF2 levels were associated with drug resistance and CSC-like properties in breast and colon cancer stem-like cells^33,34^. In order to explore the involvement of NRF2 in the ALDH1-high CSC-like cells, the levels of NRF2 and its target NQO-1 and AKR1C1 levels were examined. As shown in Fig. 3a, cellular total NRF2 level was higher in ALDH-H than that in ALDH-L cells. Higher protein and transcript levels of NQO-1 and AKR1C1 were determined in ALDH-H cells (Fig. 3a, b). We observed that most NRF2 proteins localized within the nucleus of ALDH-H cells (Fig. 3c), and ARE-driven luciferase activity was also elevated in ALDH-H cells (Fig. 3d). Whereas, there was no statistical difference in NRF2 mRNA levels between ALDH-L and ALDH-H cells (Fig. 3e). Elevated NRF2 level in ALDH-H was ALDH-dependent. When *ALDH1A1* was silenced using the specific siRNA, levels of NRF2, AKR1C1, and CSC markers were attenuated in ALDH-H (Supplementary Fig. S2, Fig. 3f, g). Additionally, the relationship between ALDH1 and NRF2 was confirmed in colon cancer cell line HCT116. When the Aldefluor-positive subpopulation was isolated as the ALDH-high fraction (Fig. 3h), levels of ALDH1A1, KLF4, BCRP, NRF2, and NQO1 were higher in ALDH-high HCT116 than those in ALDH-low HCT116 (Fig. 3i). These results indicated that NRF2 signaling activation is increased in the CSC-like ALDH-H cells.

### High NRF2 level contributes to CSC-like properties of ALDH-H cells

**Fig. 4:**
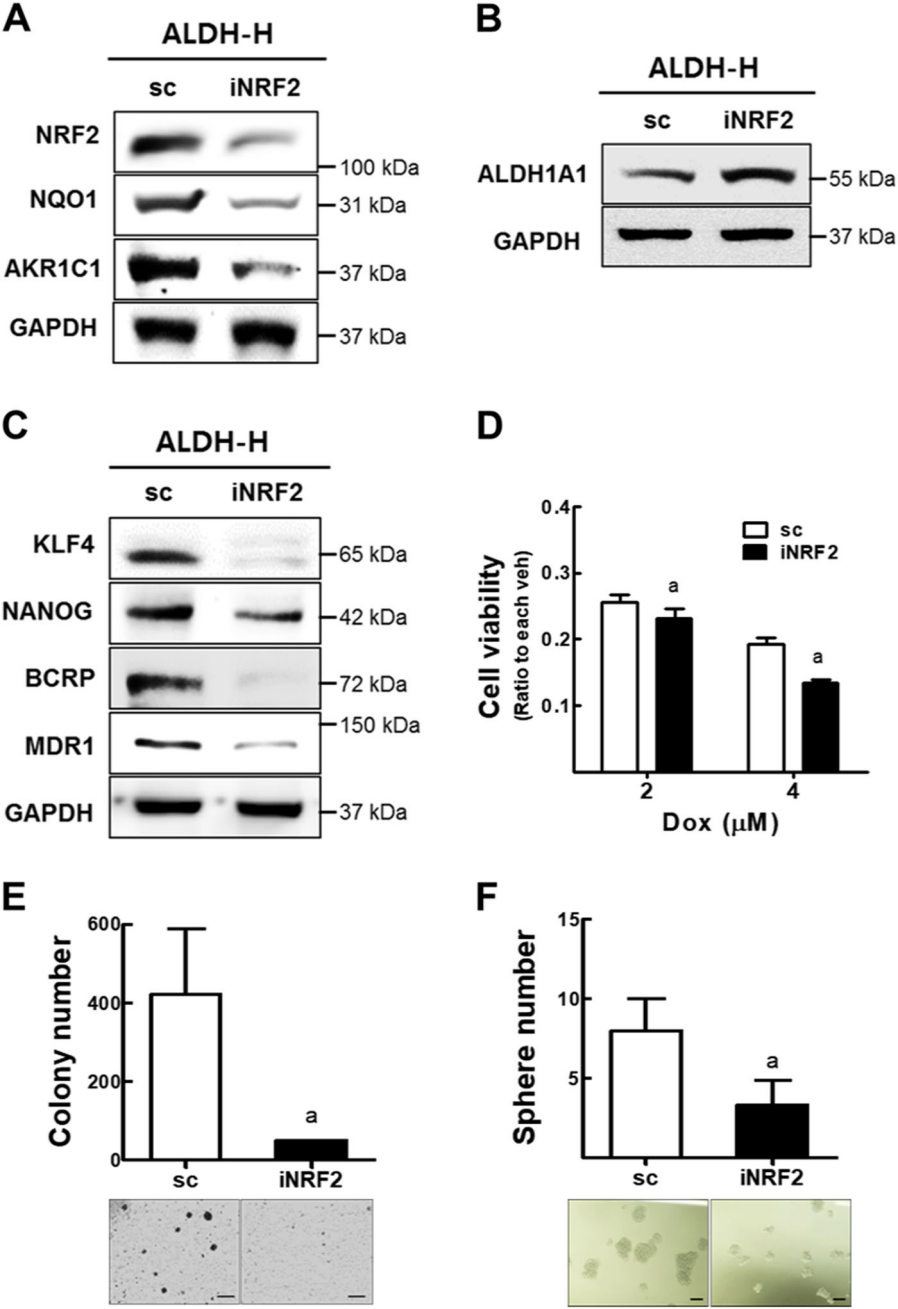
NRF2 activation is involved in CSC-like properties of ALDH-H cells. **a** Protein levels of NRF2, NQO1, and AKR1C1 were measured in established control (sc) and *NRF2*-silenced ALDH-H cells (iNRF2). **b** ALDH1A1 protein level was assessed in sc and iNRF2 ALDH-H cells. **c** Protein levels of KLF4, NANOG, BCRP, and MDR1 were determined in sc and iNRF2 ALDH-H cells. Similar blots were obtained in three independent experiments (**a**−**c**). **d** Cell viability was monitored after incubation with doxorubicin for 48 h in sc and iNRF2 ALDH-H cells. Values represent the mean ± SD from six sampled wells. ^a^*P* < 0.05 compared with vehicle-treated group. **e**−**f** Numbers of soft agar colony formation (**e**) and sphere formation (**f**) were quantified in sc and iNRF2 ALDH-H cells. Scale bar = 100 μm. Values represent mean ± SD from three independent dishes. ^a^*P* < 0.05 compared with the sc control group

In an attempt to investigate the functional involvement of NRF2 in ALDH-H cells, the effect of *NRF2*-silencing was examined. The *NRF2*-knockdown ALDH-H cells (iNRF2 ALDH-H; Supplementary Fig. S3) showed lower expression levels of NRF2, NQO1, and AKR1C1 than the control scALDH-H cells (Fig. 4a). ALDH1A1 level was not affected by *NRF2-*knockdown, which confirmed the ALDH-mediated NRF2 elevation (Fig. 4b). Levels of KLF4, NANOG, BCRP, and MDR1 were reduced in the iNRF2 ALDH-H cells (Fig. 4c). Reduced CSC markers and NRF2 expression resulted in iNRF2 ALDH-H cells more sensitive to doxorubicin cytotoxicity (Fig. 4d), and showed reduced colony/sphere-forming capacities compared to scALDH-H cells (Fig. 4e, f). These results suggested that the ALDH1A1-mediated NRF2 activation contributed to the CSC-like properties of ALDH-H cells.

### High p62 level is associated with NRF2 activation in ALDH-H cells

**Fig. 5:**
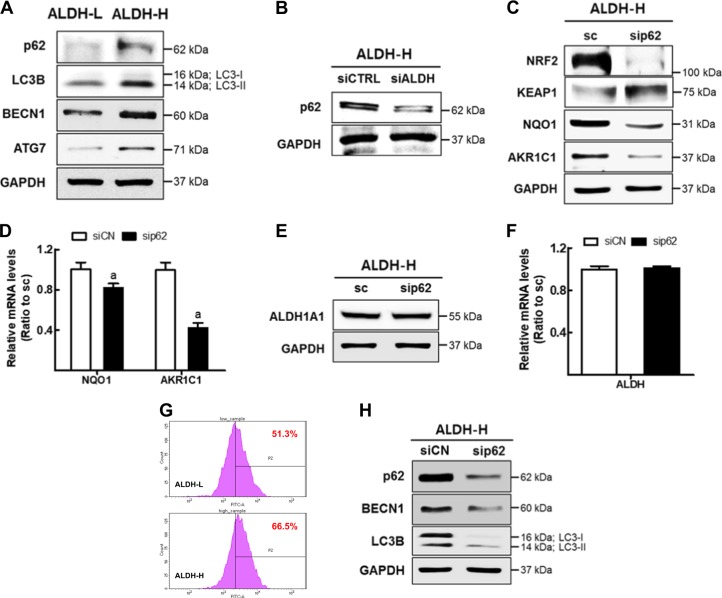
High p62 level is associated with NRF2 activation in ALDH-H cells. **a** Protein levels of p62, LC3B, BECN1, and ATG7 were measured in ALDH-L and ALDH-H cells using western blotting. **b** Protein level of p62 was determined in ALDH-H cells following nonspecific RNA (siCN) or *ALDH1A1*-specific siRNA (siALDH) transfection. **c** ALDH-H cells were transfected with *p62*-specific siRNA (sip62) or control siRNA (siCN), and protein levels of NRF2, KEAP1, NQO1, and AKR1C1 were assessed. **d** Transcript levels of *NQO1* and *AKR1C1* were determined in siCN and sip62 ALDH-H cells using RT-PCR. Values represent mean ± SD from three experiments. ^a^*P* < 0.05 compared with the siCN control group. **e** ALDH1A1 protein level was determined in siCN and sip62 ALDH-H cells. **f** Transcript level of *ALDH1A1* was assessed in siCN and sip62 ALDH-H cells. Values represent the mean ± SD from three experiments. **g** Autophagic flux was determined in ALDH-L and ALDH-H cells. Similar results were obtained in three independent experiments. **h** Western blot analysis of p62, BECN1, and LC3B was carried out in siCN and sip62 ALDH-H cells. Similar blots were obtained in three independent experiments (**a**−**c**, **e** and **h**)

As the increase in NRF2 protein level did not accompany mRNA level elevation (Fig. 3e), we speculated the involvement of p62 in NRF2 upregulation. Figure 5a shows higher levels of p62 and autophagy markers such as microtubule-associated proteins 1A/1B light chain-II (LC3B-II), Beclin 1 (BECN1), and autophagy related 7 (ATG7) in ALDH-H cells compared to ALDH-L cells. The increase of p62 was directly associated with ALDH1. Knockdown of *ALDH1A1* in ALDH-H cells diminished p62 levels (Fig. 5b). The correlation between p62 and NRF2 was demonstrated by the p62 inhibition experiment: siRNA-mediated *p62*-silencing in ALDH-H cells substantially reduced NRF2 protein level as well as its target NQO1 and AKR1C1 (Supplementary Fig. S4, Fig. 5c, d). KEAP1 level was elevated following *p62*-silencing (Fig. 5c), which confirms the p62-mediated autophagic degradation of KEAP1^37^. Whereas, ALDH1A1 levels were not affected by *p62*-knockdown, which confirms ALDH-mediated p62 upregulation (Fig. 5e, f). On the other hand, elevation of p62 seems to be associated with autophagy activation in ALDH-H cells. Autophagic flux was determined to be high in ALDH-H when compared to that in ALDH-L (Fig. 5g), and the silencing of *p62* reduced autophagy markers (BECN1 and LC3B-II) levels (Fig. 5h). These results suggested that NRF2 activation in ALDH-H CSC-like cells associated with high p62 level.

### ATRA treatment inhibits ALDH1-mediated NRF2 activation in ALDH-H cells

**Fig. 6:**
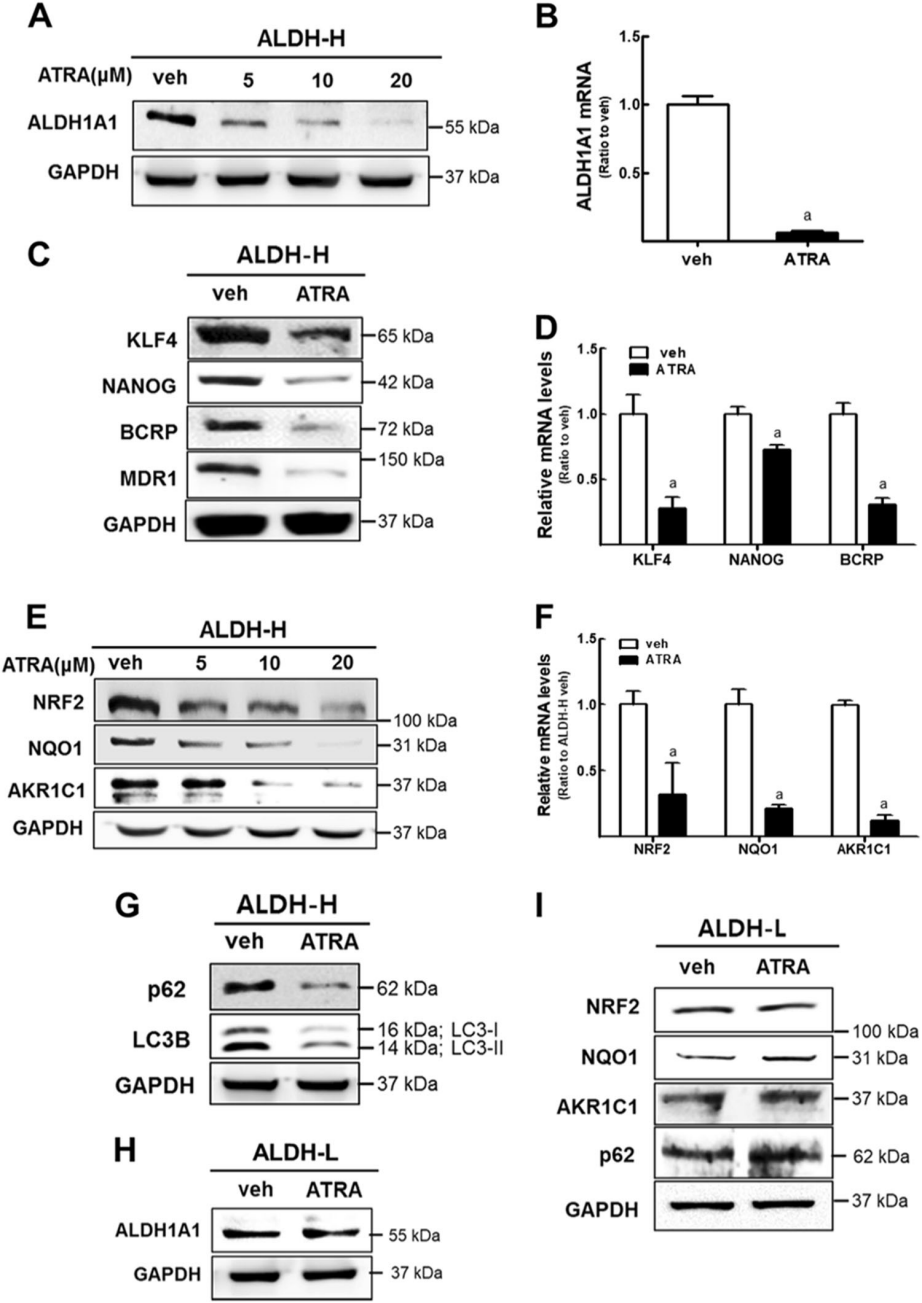
ATRA treatment inhibits NRF2 activation in ALDH-H cells. **a** After ATRA incubation for 24 h under the indicated concentration (0–20 μM), the protein level of ALDH1A1 was measured in ALDH-H cells. **b** Transcript levels of *ALDH1A1* were assessed in ALDH-H cells after ATRA treatment (10 μM, 24 h). Data represent the mean ± SD from three experiments. **c** Protein levels of KLF4, NANOG, BCRP, and MDR1 were monitored in ATRA-treated ALDH-H cells. **d** Transcript levels of *KLF4, NANOG*, and *BCRP* were measured in ATRA-treated ALDH-H. Data represent the mean ± SD from three experiments. ^a^*P* < 0.05 compared with vehicle-treated cells. **e** Protein levels of NRF2, NQO1, and AKR1C1 were determined in ATRA (5, 10, and 20 μM)-treated ALDH-H cells. **f** Transcript levels of *NRF2, NQO1*, and *AKR1C1* were measured in vehicle (veh) or ATRA-treated groups in ALDH-H cells. Data represent the mean ± SD from three experiments. ^a^*P* < 0.05 compared with vehicle-treated cells. **g** Protein level of p62 and LC3B were obtained in ATRA-treated ALDH-H cells. **h** ALDH1A1 levels were monitored in ATRA-treated ALDH-L cells. **i** Protein levels of NRF2, NQO1, AKR1C1, and p62 were measured in ATRA-treated ALDH-L cells using western blotting. All immunoblot analyses were conducted in three independent experiments and similar blots were obtained

ATRA was known to induce differentiation of leukemia cells and squamous cancer cells^38,39^. ALDH-H CSC-like ovarian cancer cells were treated with ATRA to determine its effect on ALDH1 expression. ATRA treatment (6.125−100 μM) for 24 h did not affect cell numbers in ALDH-H cells, indicating that ATRA does not influence cell proliferation within this concentration range (Supplementary Fig. S5). ATRA treatment (5−20 μM) significantly reduced protein as well as mRNA levels of ALDH1A1 (Fig. 6a, b). Levels of CSC markers KLF4 and NANOG, and drug efflux transporters BCRP and MDR1 were significantly repressed following ATRA treatment (Fig. 6c, d). These results indicate that ATRA inhibited ALDH1A1 expression presumably through differentiation induction of ALDH-H CSC-like cells. Therefore, we hypothesized that ATRA might modulate NRF2 expression as well. Indeed, the levels of NRF2, NQO1, and AKR1C1 in ALDH-H were reduced by ATRA treatment in a concentration-dependent manner (Fig. 6e, f). Levels of p62 and LC3B-II also diminished following ATRA treatment (Fig. 6g). Of note, these changes were only observed in ALDH-H CSC-like cells: ATRA treatment did not reduce ALDH1A1 level in ALDH-L cells (Fig. 6h), and NRF2 signaling and p62 level were not reduced by ATRA in ALDH-L cells (Fig. 6i). These results show that ATRA treatment repressed NRF2 signaling in ALDH-H CSC-like cells by inhibiting ALDH1 expression, which confirmed ALDH1-mediated NRF2 activation.

### ATRA suppresses CSC-like properties only in ALDH-H cells

**Fig. 7:**
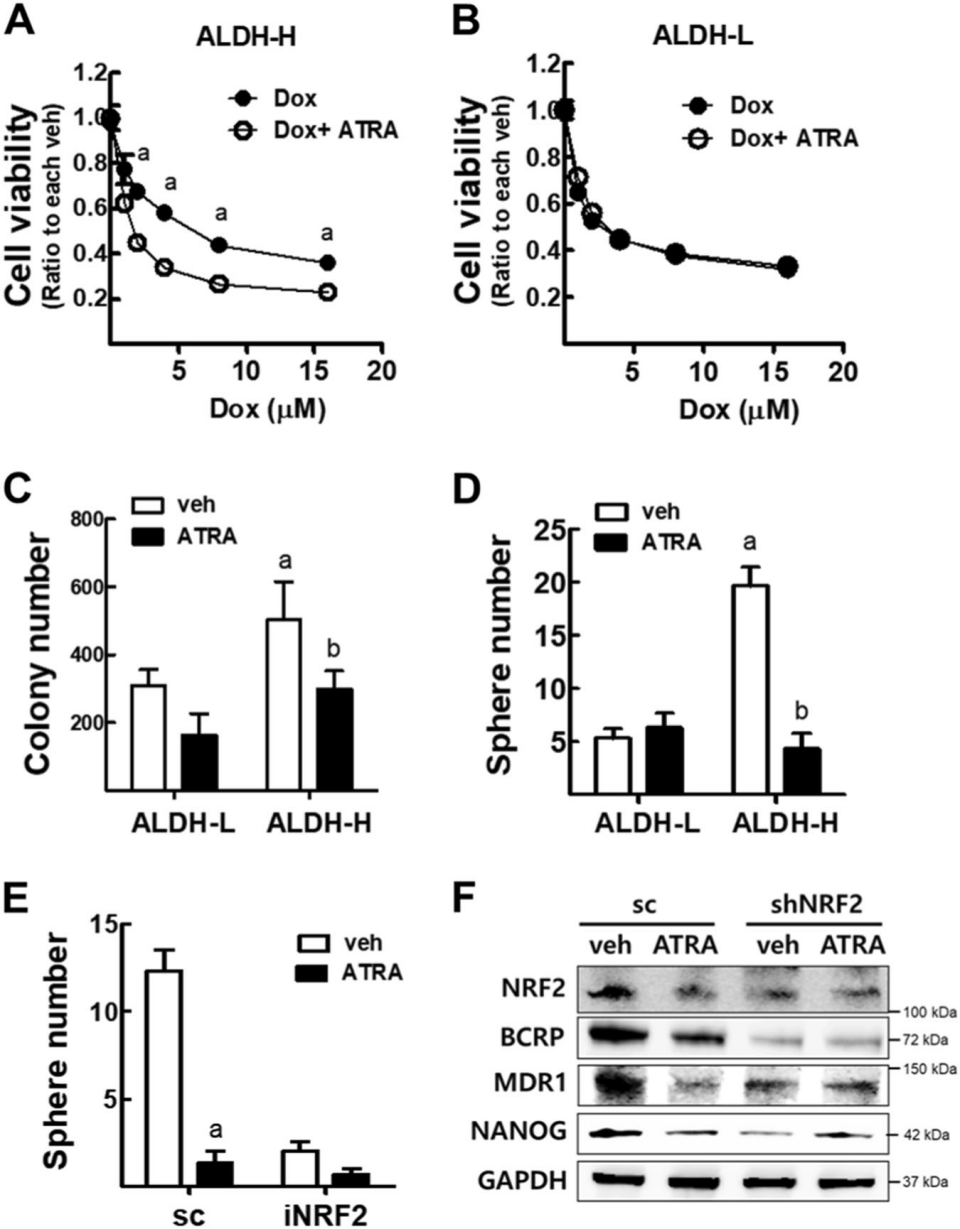
ATRA treatment reduces CSC-like properties in ALDH-H cells, but not in ALDH-L cells. **a**, **b** Cell viability after incubation with doxorubicin (Dox) for 24 h in ALDH-H (**a**) and ALDH-L cells (**b**). Values represent the mean ± SD from 8 to 10 sampled wells. ^a^*P* < 0.05 compared to the doxorubicin-treated group. **c** Soft agar colony formation was assessed in ALDH-L and ALDH-H cells after ATRA treatment (10 μM, 24 h). Values represent the mean ± SD from three experiments. ^a^*P* < 0.05 compared to vehicle-treated ALDH-L group, ^b^*P* < 0.05 compared to vehicle-treated ALDH-H group. **d** Sphere formation capacity was assessed after 3 days of sphere culture of ALDH-L and ALDH-H cells. Number of spheres (over 100 μm diameter size) was quantified. Values represent the mean ± SD from three experiments. ^a^*P* < 0.05 compared to vehicle-treated ALDH-L group, ^b^*P* < 0.05 compared to vehicle-treated ALDH-H group. **e** Sphere (over 100 μm diameter) number was quantified in sc and iNRF2 ALDH-H cells following ATRA treatment. Values represent the mean ± SD from three experiments. ^a^*P* < 0.05 compared to each vehicle-treated group. **f** Protein levels of NRF2, BCRP, MDR1, and NANOG were measured in sc and *NRF2*-silenced ALDH-H cells following ATRA treatment. Similar blot were obtained in three independent experiments

Since ATRA treatment was found to downregulate the expression of CSC markers and NRF2 in ALDH-H cells, we explored the functional involvement of ATRA in CSC-like properties of ALDH-H cells. First, coincubation of ATRA and doxorubicin in ALDH-H (Fig. 7a) and ALDH-L cells (Fig. 7b) showed that only ALDH-H cells become more susceptible to doxorubicin toxicity than doxorubicin-alone treatment. Second, ATRA treatment significantly inhibited colony and sphere formation capacities in ALDH-H cells but not in ALDH-L cells (Fig. 7c, d). Third, the effect of ATRA on CSC-like properties was associated with NRF2. The inhibitory effect of ATRA on sphere formation in ALDH-H was blunted when NRF2 expression was silenced (Fig. 7e), and the effects of ATRA on NRF2 and CSC markers were not observed in *NRF2*-knockdown ALDH-H cells (Fig. 7f). These results indicate that ATRA suppressed CSC-like properties of ALDH-H by inhibiting NRF2 signaling.

### ATRA inhibits ALDH-H-derived tumor growth

**Fig. 8:**
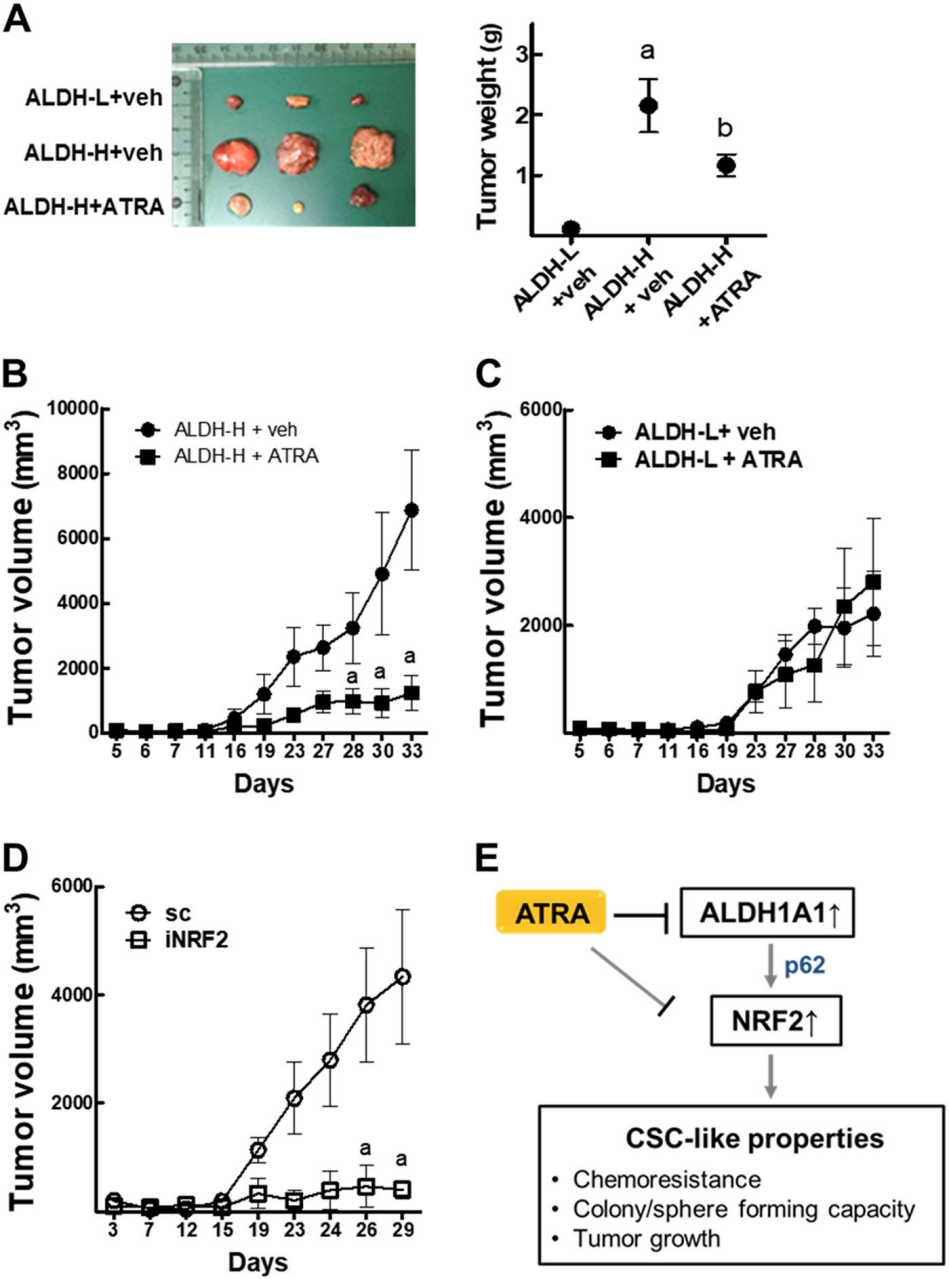
ATRA treatment inhibits the ALDH-H-derived tumor growth. **a** ALDH-L and ALDH-H cells were implanted in nude mice and tumor volume was measured every week from 5 to 33 weeks after inoculation. When tumor size reached 100−200 mm^3^, mice received vehicle (veh) or ATRA (10 mg/kg) three times a week. Tumors (*n* = 4–5) were obtained from mouse xenografts (left) and tumor weight was measured (right). **b** Tumor volume assessed in ALDH-H inoculated mice (*n* = 4–5). Mice received vehicle (veh) or ATRA (10 mg/kg). **c** Tumor volume assessed in ALDH-L inoculated mice (*n* = 4–5). Mice received vehicle (veh) or ATRA (10 mg/kg). **d** Tumor volume assessed in sc and iNRF2 ALDH-H inoculated mice (*n* = 3). ^a^*P* < 0.05 compared to each vehicle (veh)-treated group. **e** The p62-associated NRF2 activation contributes to CSC-like properties of ALDH1A1-high ovarian cancer cells. ATRA treatment suppresses ALDH1-mediated NRF2 activation and exhibits inhibitory effects on CSC-like properties. ATRA can directly inhibit NRF2 transcription activity as well

ALDH-L and ALDH-H cells inoculated in BALB/c-*nu/nu* mice showed results similar to in vitro results. The growth of ALDH-H-derived tumors was significantly higher than that of ALDH-L cells, and ATRA treatment (10 mg/kg, three times a week) suppressed tumor growth (Fig. 8a, b). Whereas, ATRA treatment did not show tumor inhibitory effects in the ALDH-L group (Fig. 8c). Tumors bearing *NRF2*-knockdown ALDH-H cells showed significant growth retardation when compared to the sc control, which confirms the critical role of NRF2 in tumor growth of ALDH-H cells (Fig. 8d).

## Discussion

ALDHs, enzymes that oxidize endogenous as well as exogenous aldehydes using NAD(P)+, have been suggested as one of the major CSC markers^11,12^. High ALDH activity is attributed to the increased expression of ALDH1A1 in many types of cancer cells; therefore, ALDH1A1 isozyme is used as a marker for the enrichment of CSC subpopulations from tumors and cancer cell lines. The link between ALDH1 and CSC-like properties can be supported by experimental ALDH1 inhibition. The interfering RNA-mediated *ALDH1*-silencing sensitized drug-resistant ovarian CSCs to taxane and platinum therapy^40^. Silencing of *ALDH1A1* enhanced gemcitabine-induced apoptosis in pancreatic cancer cells^41^. The treatment of drug-tolerant cancer cells with ALDH inhibitor disulfiram elevated cellular ROS to cytotoxic levels and induced cell death^42^. These reports indicate the critical role of ALDH1 in the development of CSC-like properties of cancer cells. As clinical evidence, ALDH1A1 expression has been associated with poor clinical outcomes in ovarian cancer patients^43–45^.

Several explanations have been suggested for the mechanism by which ALDH1 mediates CSC-like properties such as chemoresistance and stress resistance. First, ALDH1 directly involves the metabolism of specific alkylating anticancer agents, such as cyclophosphamide, and inhibits cytotoxic metabolites production, resulting in resistance to these alkylating drugs^46^. Second, ALDH1 was shown to contribute to cell protection from aldehyde-induced toxicity by removing reactive aldehydes such as 4-hydroxy-2-nonenal^47^. Third, ALDH1 can participate in ROS reduction by generating NAD(P)H, a major cellular reducing factor for GSH regeneration^47^. Particularly, the antioxidant effect of ALDH1 has been suggested as an underlying mechanism of resistance of ALDH1-high cancer cells to oxidative stress-associated chemotherapeutic drugs such as taxanes and platinums^48^. It is noteworthy that ALDH-mediated chemoresistance was not confined to specific drugs. ALDH1-enriched subpopulations of cancer cells exhibited resistance to multiple drugs including doxorubicin, cisplatin, taxanes, 5-fluorouracil (5-FU), cytarabine, and gemcitabine, which raises the potential association of drug efflux transporters with ALDH1 overexpression^48^. Indeed, the treatment of drug-resistant ovarian cancer cells with diethylaminobenzaldehyde (DEAB), an inhibitor of ALDH1, downregulated BCRP and MDR1 levels with concomitant elevation in drug sensitivity^21^. As a molecular basis of ALDH1-mediated chemoresistance and CSC-like properties, our study revealed the involvement of NRF2 signaling. We observed that levels of NRF2 and its target NOQ1 and AKR1C1 were significantly elevated in ALDH1A1-high ovarian CSC-like cells, and the increase in p62 was related to ALDH1-mediated NRF2 upregulation. As a functional implication of NRF2 activation, high levels of BCRP and MDR1 in ALDH-H cells were reduced by *NRF2* knockdown. CSC-like phenotypes such as chemoresistance, colony/sphere formation, and tumor growth, as well as CSC marker expression were significantly blocked in *NRF2*-silenced ALDH-H cells. Furthermore, we demonstrated that ATRA treatment suppressed the ALDH1A1-p62 axis, and thereby inhibited NRF2 activation, resulting in the attenuation of CSC-like properties only in ALDH1-high ovarian cancer cells. These results provide a new basis for the molecular mechanism of the induction of CSC characteristics by ALDH1.

The association of NRF2 signaling in CSC has been indicated by several recent reports. The prolonged incubation of breast cancer cells with anticancer drugs generated CSC-like cells with low ROS levels, and elevated GPX, SOD, and NRF2 levels^49^. In the colon CSC-like cells, the secretome was found to contain high levels of NRF2-target antioxidant and detoxifying proteins^50^. *NRF2* silencing blocked self-renewal capacities in glioblastoma CSC-like cells^32^, and NRF2-mediated drug resistance was demonstrated in sphere-cultured breast cancer cells^51^. We previously showed that NRF2 level increased in spheroids of breast and colon cancer cells, and NRF2 elevation was responsible for drug efflux transporter expression, chemoresistance, and spheroid growth^33,34^. Additionally, a recent study has shown that NRF2 level was increased by CD44-p62 signaling in CD44+ high breast CSC-like cells, and the coexpression of CD44 and NRF2 was confirmed in clinical samples of breast cancers^52^. These reports indicate the involvement of NRF2 in CSC-like properties of multiple types of CSCs, suggesting the potential role of NRF2 as a common factor leading to the development of stress resistance-related characteristics of CSCs.

Our study indicated that upregulation of autophagy-associated p62 was responsible for NRF2 activation in ALDH1A1-high CSC-like cells. The involvement of p62-related signaling in CSC maintenance has been demonstrated in several types of cancer cells. In glioblastoma CSCs, the autophagy-associated factors DRAM1 and p62 were highly expressed, and subsequent activation of mitogen-activated protein kinase and c-MET had a role in cell migration and invasion^53^. High p62 level was found in spheroid, CD44^high^CD24^low^ subpopulation, side population, and ALDH1-positive subpopulation of breast CSC-like cells, and p62 was associated with CSC-related gene expression through *MYC* elevation^54^. High levels of p62 in mice led to NRF2 activation and protected hepatocellular carcinoma (HCC)-initiating cells from ROS, which resulted in HCC induction^55^. Similarly, we previously observed that p62 was accumulated in spheroid breast cancer cells, and the inhibition of p62 blocked NRF2 elevation^33^. These reports suggest that p62 elevation is a molecular link that leads to the NRF2-mediated stress resistance of CSC-like cells; however, at this time point, the mechanism of p62 elevation in CSC-like cells remains unclear. On the other hand, there is a possibility that increased p62 is related with autophagy activation in ALDH-H cells. Levels of autophagy-associated factors, including BECN1, ATG7, and LC3B-II, were significantly higher in ALDH1-high CSC-like cells, and the silencing of p62 reduced the levels of autophagy markers. These indicate that autophagy might be essential for adaptive survival of CSCs. Since CSCs are located in the microenvironment of hypoxia and nutrient depletion, catabolic process-favoring metabolic adaptation would be required for ATP generation^56^. Indeed, autophagy-associated BECN1 level was increased in the ALDH1A1-high subpopulation from mammospheres^57^. Paclitaxel resistance of CD44-high colorectal CSCs was attributed to autophagy activation^58^. The inhibition of autophagy suppressed spheroid formation in breast CSCs^59^, and autophagy activation mediated pancreatic CSCs survival under hypoxic environment^60^.

Presumably through the induction of cell differentiation and inhibition of CSC stemness, ATRA treatment showed chemosensitization effects^61^. Lung cancer cells with high ALDH1 activity treated with ATRA decreased ALDH1A1 protein levels without affecting mRNA levels, resulting in sensitization to 4-hydroperoxycyclophosphamide^25^. ATRA reduced ALDH1A1 levels and inhibited sphere formation and invasion in ALDH1-high cisplatin-resistant ovarian cancer cells^62^. Similarly, the inhibitory effect of ATRA on ALDH1 was confirmed in our system: ATRA effectively reduced ALDH1A1 expression and inhibited CSC marker expression, tumor growth, and colony/sphere formation. Additionally, we showed that ATRA inhibited NRF2 activation as a downstream event of ALDH1 suppression and in particular, we demonstrated that the effect of ATRA on ALDH1A1-NRF2 axis was restricted to ALDH1-high cells. ATRA treatment did not diminish doxorubicin resistance, colony/sphere formation, and tumor growth in ALDH1-low doxorubicin-resistant cells. Moreover, ATRA did not inhibit sphere formation of ALDH1-high cells when *NRF2* was silenced. These results suggest that NRF2 suppression could be one of the molecular mechanisms of ATRA-mediated inhibition of CSC-like properties, and the effect of ATRA on NRF2 could be ALDH-associated CSC-specific phenomenon. It is also noteworthy that ATRA has been demonstrated to diminished NRF2 binding to the ARE by enhancing complex formation of NRF2 with retinoic acid receptor-α^63^. Although the ALDH1 inhibition-mediated NRF2 suppression seems to be the primary mode of action of ATRA in our system, this additional suppressive action on NRF2 could be beneficial to control CSC resistance.

Overall, our study demonstrated that high ALDH1A1 led to NRF2 activation via p62-associated pathway in ALDH1-high CSC-like ovarian cancer cells (Fig. 8e). NRF2 activation in these cells contributed to CSC-like properties such as drug resistance, colony/sphere formation, tumor growth, and high stemness marker expression. The functional involvement of NRF2 in CSC-like properties could be confirmed by ATRA effects. ATRA suppressed CSC-like properties only in ALDH1-high cancer cells by inhibiting the ALDH1-mediated NRF2 activation, further suggesting the molecular basis of ATRA effect in CSCs.

## Materials and methods

### Reagents

The antibody against ALDH1A1 was obtained from Abcam (Cambridge, UK). Antibodies for NRF2, NQO1, BECN1, ATG7, lamin B, and glyceraldehyde 3-phosphate dehydrogenase (GAPDH) were from Santa Cruz Biotechnology (Santa Cruz, CA, USA). Antibodies recognizing BCRP, KLF4, NANOG, c-Met, p62, LC3B, and MDR1 were purchased from Cell Signaling Technology (Danvers, MA, USA). AKR1C1 antibody was purchased from Abnova (Taipei City, Taiwan). The lentiviral expression plasmid for human NRF2 short hairpin RNA (shRNA), MISSION^TM^ Lentiviral Packaging Mix, hexadimethrine bromide, doxorubicin hydrochloride, ATRA, puromycin, and 3-(4,5-dimethylthiazol-2-yl)−2,5-diphenyltetrazolium bromide (MTT) were from Sigma-Aldrich (Saint Louis, MO, USA). The luciferase reporter plasmid for ARE was a gift from Dr. Wakabayashi (University of Pittsburg, PA, USA). The SYBR Premix ExTaq system was obtained from Takara (Otsu, Japan). Matrigel Basement Membrane Matrix was purchased from Corning Costar Corp (Cambridge, MA, USA).

### Cell culture

The human ovarian cancer cell line A2780 was obtained from the European Collection of Cell Cultures (Salisbury, Wiltshire, UK). A2780DR was established in our previous study^35^. Cells were maintained in RPMI1640 (Hyclone, Logan, UT, USA) supplemented with 10% fetal bovine serum (Corning Costar Corp., Cambridge, MA, USA) and 1% penicillin/streptomycin (WelGENE Inc., Daegu, Republic of Korea). The cells were grown at 37 °C in a humidified 5% CO_2_ atmosphere. All cell lines used in this study were routinely checked for mycoplasma contamination (MycoAlert Mycoplasma Detection Kit, Lonza, Allendale, NJ, USA).

### Establishment of ALDH-H and ALDH-L cells

For the isolation of ALDH1-high subpopulation, ALDH1 activity was assessed using an Aldefluor kit (Stem Cell Technologies, Vancouver, British Columbia, Canada). Approximately 1×10^6^ A2780DR cells were harvested and incubated in Aldefluor assay buffer containing a 2.0 μM ALDH1A1 substrate for 40 min at 37 °C. Negative control samples were treated with 50 μM of DEAB as an inhibitor of ALDH1 enzymatic activity. The fluorescence intensity of the stained cells was analyzed by using a FACS AriaIII Cell Sorter Flow Cytometer (BD Biosciences, Franklin Lakes, NJ, USA). The DEAB-treated cells were used to define the baseline. Based on the fluorescence intensity below or beyond the threshold defined by the reaction with DEAB, each of the sorted cells was designated to be “ALDH1-low (ALDH-L)” or “ALDH1-high (ALDH-H)” cells.

### Establishment of *NRF2*-knockdown ALDH-H cells

To establish the *NRF2*-knockdown ALDH-H cell line (iNRF2), ALDH-H cells were incubated with lentiviral particles containing either the nonspecific scrambled RNA (scRNA) plasmid (pLKO.1-scRNA, sc) or *NRF2*-specific shRNA (5′-CCGGGCTCCTACTGTGATGTGAAATCTCGAGATTTCACATCACAGTAGGAGCTTTTT-3′) plasmid (pLKO.1-NRF2 shRNA, iNRF2) in the presence of hexadimethrine bromide (8 mg/mL). After 48 h incubation, viral particle-containing medium was removed and cells were recovered in the fresh medium for 24 h. Cells were subsequently placed under puromycin (2 μg/mL) for the selection of stable transgene-expressing cells for up to 4 weeks.

### siRNAs transfection

Predesigned ALDH1A1 siRNA (forward: 5′-GAGAGUACGGUUUCCAUGA-3′, reverse: 5′-UCAUGGAAACCGUACUCUC-3′) and a scrambled control siRNA^36,52^ were purchased from the Bioneer Corporation (Daejeon, Republic of Korea). For transfection, 5×10^5^ ALDH-H cells in 60-mm plates were incubated in Opti-MEM media (Hyclone, Logan, UT, USA) and transfected with the siRNAs using a Lipofectamine 2000 reagent (Life Technologies, Carlsbad, CA, USA). Predesigned p62 siRNA was transfected into ALDH-H cells as described previously^33^.

### Total RNA extraction and real-time reverse transcriptase (RT)-polymerase chain reaction (PCR)

Total RNAs were isolated from the seeded cells in 100-mm^2^ plates using the TRIzol reagent (Thermo Fisher Scientific Inc., Waltham, MA, USA) and processed for the synthesis of cDNAs. RT reactions were performed by incubating 200 ng of the total RNAs with a reaction mixture, which contained 0.5 μg/μL oligo dT_12–18_ and 200 U/μL Moloney murine leukemia virus RT (Life Technologies). Real-time RT-PCR was carried out using a Roche Light Cycler (Mannheim, Germany) with the Takara SYBR Premix ExTaq System for relative quantification. Primers were synthesized by the Bioneer Cooperation, and the primer sequences for the human *NRF2, AKR1C1, NQO1, ALDH1A1, NANOG, KLF4, BCRP, SOX2*, and *HPRT1* are indicated in Table 2.

**Table 2:** Forward and reverse primer sequence for RT-PCR analysis of each mRNA level

Gene	Forward primer	Reverse primer
*NRF2*	5′-ATAGCTGAGCCCAGTATC-3′	5′-CATGCACGTGAGTGCTCT-3′
*AKR1C1*	5′-CGAGAAGAACCATGGGTGGA-3′	5′-GGCCACAAA-GGACTGGGTCC-3′
*NQO1*	5′- CAGTGGTTTGGAGTCCCTGCC-3′	5′-TCCCCGTGGATCCCTTGCAG-3′
*ALDH1A1*	5′-TCCACATTCCAGTTTGGCCC-3′	5′-TTCGAAGGAGTGTTGAGCG-3′
*NANOG*	5′-AGGCCTTCTGCGTCACACCATTG-3′	5′-CAGCCTCCAGCAGATGCAAGAAC-3′
*KLF4*	5′-GGCGAATTTCCATCCACAGCCG-3′	5′-ACACTTGTGATTACGCGGGCTGC-3′
*BCRP*	5′-CACAACCATTGCATCTTGGCTG-3′	5′-TGAGAGATCGATGCCCTGCTTT-3′
*SOX2*	5′-GCTCGCCATGCTATTGCCG-3′	5′-CATGAAGGAGCACCCGGATTA-3′
*OCT4*	5′-TGGCTGATCTGCTGCAGTGTG-3′	5′-TGCAGAAGTGGGTGGAGGAAGC-3′
*HPRT1*	5′-TGGCGTCGTGATTAGTGATG-3′	5′-GCTACAATGTG-ATGGCCTCC-3′

### Cell number count

To determine viable cell numbers, cells were plated at a density of 5×10^3^ cells/well in 96-well plates. Cells were incubated with varied concentrations of doxorubicin for 24 h, and MTT solution (2 mg/mL) was added for a further 3-h-incubation. The MTT solution was removed, 100 μL/well of dimethyl sulfoxide was added, and the absorbance was measured at 540 nm using a SPECTRO Star^Nano^ (BMG LABTECH GmbH, Ortenberg, Germany). Cell number was also directly counted using a TC10 Automated Cell Counter (Bio-Rad Laboratories, Inc., Hercules, CA, USA).

### Immunoblot analysis

Cells were lysed with radioimmunoprecipitation assay lysis buffer containing a protease inhibitor cocktail (Sigma-Aldrich, Saint Louis, MO, USA). The protein concentration was determined using a bicinchoninic acid assay kit (Thermo Fisher Scientific Inc.). Quantified protein samples were separated by electrophoresis on 6−15% sodium dodecyl sulfate–polyacrylamide gels and transferred to nitrocellulose membranes (GE Whatman, Dassel, Germany). The membrane was blocked with 3% bovine serum albumin for 1 h and incubated with the primary antibody overnight. The next day, the membrane was washed with Tween 20-containing phosphate buffered saline (PBS), and incubated with the corresponding secondary antibody for 1 h. Following the addition of the enhanced chemiluminescence reagent (Thermo Fisher Scientific Inc.), images were detected using a GE Healthcare LAS-4000 mini imager (GE Healthcare Life Sciences, Piscataway, NJ, USA).

### Sphere formation assay

Cells were plated at a density of 1×10^3^ cells in 96-well ultralow attachment plates (Corning Costar Corp., Cambridge, MA, USA), and were incubated with a serum-free RPMI1640 and Nutrient Mixture F-12 medium supplemented with 20 ng/mL basic fibroblast growth factor (R&D System, Minneapolis, MN, USA), 20 ng/mL epithelial growth factor, B27 (1:50, Life Technologies), 0.5 μg/mL hydrocortisone (Sigma-Aldrich, St. Louis, MO, USA), 5 μg/mL bovine insulin (Cell Application Inc., San Diego, CA, USA), and 1% penicillin/streptomycin (HyClone, Logan, UT, USA) as described previously^33^. In the sphere culture condition, cells were grown for 3 days and spheroids were monitored using a Carl Zeiss Primovert Microscopy (Carl-Zeiss, Germany).

### Soft agar colony forming assay

To evaluate the anchorage-independent growth ability, soft agar colony formation assay was performed. Cells (5×10^3^) were suspended in top soft agar layer (0.35%) and seeded into the 0.5% base agar-coated six-well plates. Colonies were allowed to grow for 2−3 weeks and number of colonies was counted using an ECLIPSE Ti inverted microscope and the NIS-Elements AR (V. 4.0) computer software program (NIKON Instruments Korea, Gangnam, Seoul, Korea) as described previously^52^.

### Tumor xenografts

The ALDH-H, ALDH-L, scALDH-H, NRF2i ALDH-H cells were harvested and suspended with equal volumes of PBS and Matrigel. A suspension with 5×10^6^ cells in 0.1 mL Matrigel mixture was injected subcutaneously into the flank of 8-week-old BALB/c-*nu/nu* mice (Orient Bio Inc., Gyeonggi-do, Republic of Korea). The tumor growth was monitored weekly using calipers and tumor volume was calculated by the formula *V* = (*a*^2^ × *b*)/2, where a and *b* are the width and the length in millimeters, respectively. When tumor size reached 100−200 mm^3^, mice received vehicle or ATRA (10 mg/kg) three times a week for 4 weeks. Each group contained 4−5 animals. The animal experiment was approved by the Institutional Ethics Committee on Animal Care and Experimentation at the Catholic University of Korea (approval number: 2016-035-01).

### Cyto-ID autophagy detection

Autophagic flux was determined in live cells using Cyto-ID autophagy detection kit as described previously^52^. Briefly, cells in 60-mm plates were stained with Cyto-ID dye according to the manufacturer’s instructions and autophagic vacuoles were measured using a 488 nm laser source in a Becton-Dickinson FACSCanto and data were analyzed with FACSDiva software (Becton-Dickinson, San Jose, CA, USA).

### Statistical analysis

Statistical significance was determined with Student’s *t* tests or one-way analyses of variance (ANOVA), followed by Student−Newman−Keuls tests for multiple comparisons. These analyses were conducted with GraphPad Prism software (GraphPad Software Inc., La Jolla, CA).

## Electronic supplementary material

Fig. S1 - S5
