# Supplementary material for: High NRF2 level mediates cancer stem cell-like properties of aldehyde dehydrogenase (ALDH)-high ovarian cancer cells: inhibitory role of all-trans retinoic acid in ALDH/NRF2 signaling
Source: Cell Death Dis. 2018 Aug 30;9(9):896. doi: 10.1038/s41419-018-0903-4 (PMC6117306; doi:10.1038/s41419-018-0903-4)
Supplement: Supplementary file 1 — Fig. S1 - S5 [file 41419_2018_903_MOESM1_ESM.docx]

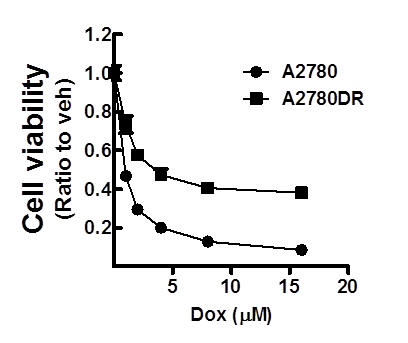


Supplementary Fig. S1. Viability of parental A2780 and A2780DR was assessed following doxorubicin incubation for 24 h.


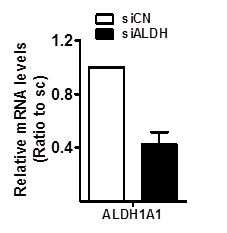


Supplementary Fig. S2. *ALDH1A1*-specific siRNA (iALDH) was transfected into ALDH-H cells and *ALDH1A1* transcript level was determined by using RT-PCR analysis. Nonspecific scrambled RNA (sc) was used as a negative control.


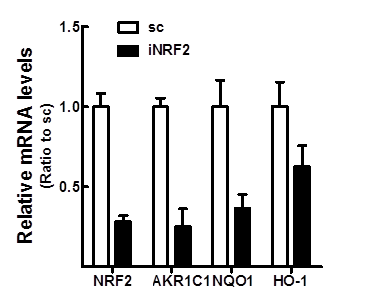


Supplementary Fig. S3. Transcript levels for NRF2, AKR1C1, NQO1, and heme oxygenase-1 (HO-1) were determined in the control (sc) and *NRF2*-knockdown stable cell line (iNRF2).


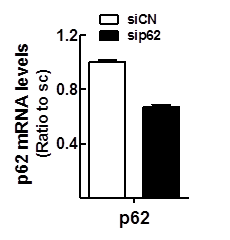


Supplementary Fig. S4. Transcript level for p62 was assessed following transfection of ALDH-H cells with nonspecific siRNA (siCN) or p62-specific siRNA (sip62).


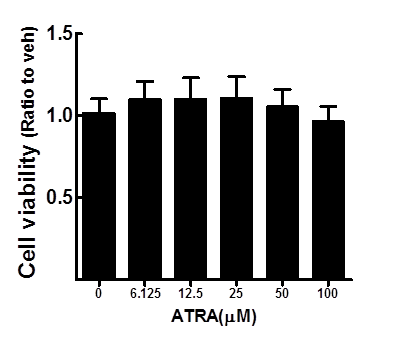


Supplementary Fig. S5. Cell number was counted using MTT analysis following the incubation of ALDH-H cells with ATRA (6.125−100 μM, 24 h)
